# Civil Hospitals and Dispensaries in Madras Presidency

**Published:** 1899-07-22

**Authors:** 


### CIVIL HOSPITALS AND DISPENSARIES IN MADRAS PRESIDENCY.

Both the town and the Presidency of Madras are to be congratulated on the effective manner in which they fought the Plague during 1897. One case was imported into the town. A syce, who came from Bombay to attend the Guindy Races, was taken ill and conveyed to the General Hospital, where he was admitted "with doubtful symptoms." The disease developed, proved fatal, and there was a post-mortem examination. The resident medical officer who performed it, and one toty who served in the dissecting-room, were subsequently attacked. The former recovered, the latter died. Precautions against the spread of infection were strictly carried out, and no further cases occurred. Therefore the mortality from plague for the year is two only.

The record for the whole of the Presidency is equally satisfactory. Plague inspection stations were opened at five different centres during the year. A retired military assistant surgeon was placed in charge of each, with an adequate subordinate medical and menial staff to carry out the regulations; 4,721 trains were inspected, and a daily average of 3,313 passengers examined; 27,231 persons were noted as passing through plague-infected areas, and of these 676 were detained for observation. Only two cases of plague came under treatment, one proved fatal, the other, being attended to sufficiently early, recovered. But all these precautionary measures caused a heavy drain upon the medical resources of the Presidency, weakened by the war on the frontier, which had necessitated the withdrawal of thirteen medical officers and military assistant surgeons. Arrangements had to be made to fill the vacancies thus created, and the services of senior departmental civil assistant surgeons, temporarily retired medical assistant surgeons, and private practitioners possessing medical degrees, were employed. To secure a sufficient number of subordinates of the hospital assistant class very liberal terms had to be offered as an inducement, and this materially increased the total expenditure for the year. In 1896 it was Rs.10,07,079, in 1897 Rs.10,49,605. It is satisfactory to find that of this Rs.15,668 was contributed from mission funds and Rs.44,290 from subscriptions direct to the mission hospital. Another pleasant feature is the much larger amount which was spent on bazaar medicines during 1897 than in the previous year, showing that the encouragement which has been given, wherever possible, to the use of indigenous, in preference to European drugs, is having the desired effect. Waring's book on "Bazaar Medicines" has been supplied to all medical institutions. The number of beds occupied in the civil hospitals and dispensaries in the Presidency of Madras is still comparatively small, 57 per cent. only, but the percentage continues to rise, showing undoubtedly the growing popularity of European, as opposed to native, methods of medical treatment.

